# Adverse Effects and Precautionary Measures for Isotretinoin Use in Patients with Acne Vulgaris: A Single-Center Study

**DOI:** 10.3390/healthcare13131617

**Published:** 2025-07-07

**Authors:** Mohammed Saif Anaam, Dalia A. AlShibl, Saeed Alfadly, Munirah Yousef Aloyuni, Fawaz Hamdan Al Harbi, Hussam Alhmoud, Ibrahim S. Alhomoud, Waleed Mohammad Altowayan

**Affiliations:** 1 Department of Pharmacy Practice, College of Pharmacy, Qassim University, Buraydah 51452, Saudi Arabia; m.anaam@qu.edu.sa (M.S.A.); s.alfadly@qu.edu.sa (S.A.); h.alhmoud@qu.edu.sa (H.A.); w.altowayan@qu.edu.sa (W.M.A.); 2 Pharmacy Department, King Fahad Hospital of the University, Imam Abdulrahman Bin Faisal University, Dammam 34221, Saudi Arabia; alshibldaliaa@gmail.com; 3 College of Pharmacy, Qassim University, Buraydah 51452, Saudi Arabia; munirah.yousef.a@gmail.com; 4 Department of Pharmacy, King Saud Hospital, Unaizah 56249, Saudi Arabia; falharbi22@moh.gov.sa

**Keywords:** isotretinoin, teratogenicity, acne vulgaris, knowledge, questionnaire, adverse effects

## Abstract

Background: Oral isotretinoin is an effective treatment for refractory and moderate acne unresponsive to conventional therapies, considered the most effective option for such cases. Objective: This study aimed to evaluate the knowledge, concerns, and experiences of acne patients undergoing isotretinoin treatment in Qassim, Saudi Arabia, with a focus on commonly reported adverse effects. Methods: A cross-sectional study was conducted from December 2023 to February 2024 using a self-administered questionnaire. This study targeted male and female acne vulgaris patients from the Qassim region attending the outpatient dermatology clinic at King Saud Hospital (KSH). Results: A total of 131 acne patients participated. Of these, 97.7% had heard of isotretinoin, and 92.4% were aware of its side effects. The most common sources of information were colleagues, friends, or family (37.4%), followed by previous use (26%) and healthcare professionals (24%). The most frequently reported side effect was dryness (51.9%), followed by liver function changes (24.4%) and fetal abnormalities (13%). There was a significant association between educational level and knowledge of isotretinoin’s side effects (*p* = 0.003) and awareness of specific side effects (*p* < 0.001). Conclusion: Most acne patients had sufficient knowledge of isotretinoin and its adverse effects, with dryness being the most commonly reported side effect. The primary sources of information were non-medical, highlighting the need for health education to ensure informed and safe isotretinoin use.

## 1. Introduction

Acne vulgaris is a chronic inflammatory skin disorder [1]. Acne vulgaris affects approximately 117.4 million people worldwide [2]. An estimated 85% of individuals aged 12–24 are impacted by acne vulgaris [1,3]. In Saudi Arabia, some studies have reported a high prevalence of acne among adolescents and young adults, ranging from 56% to 71% [4,5,6]. A U.S. survey of over 1000 adults found that 43% of males and 51% of females self-reported acne in their 20s years. However, these rates declined by age 50 and older to 7% and 15%, respectively [7]. Acne affects all age groups but is commonly prevalent among adolescents by approximately 85% [2,3]. The prevalence of acne declines by age 50 and older to 7% and 15%, respectively [7]. Acne vulgaris is characterized by closed or open comedones, papules, pustules, or nodules [1]. Acne vulgaris primarily involves the face and trunk and may cause pain, redness, pigmentation changes, or scar formation [1]. The development of acne involves multiple factors, including increased sebum production, hormone dysregulation, stress, diet, microbial colonization with *Cutibacterium acnes*, follicular hyperkeratinization, and inflammation [1,8]. Although no standard grading system has been established for classification, a widely acceptable approach is to classify acne according to severity as mild, moderate, or severe [8]. Acne can cause a significant burden to individuals, as it is identified as the second most impactful skin disorder based on disability-adjusted life years [9]. It can also impact quality of life significantly to a similar extent as other chronic conditions such as asthma and arthritis [10]. Acne vulgaris can also lead to significant psychological consequences, including low self-esteem, anxiety, and depression [11,12].

The mainstays of acne treatment have remained primarily unchanged over recent years [13]. Treatment options include topical and systemic therapies. The treatment regimen selection is based on several factors including the severity of acne, regions affected, patient preferences, potential adverse effects, and cost. Isotretinoin is derived from vitamin A and was introduced to the market in 1982 [14]. The mechanism of isotretinoin functions mainly by reducing the size of sebaceous glands and decreasing sebum production, with a half-life of 10–20 h [14]. Because of its significant risk of severe fetal malformations, isotretinoin use is strictly contraindicated in women of childbearing potential unless stringent contraceptive measures are implemented [14]. Oral isotretinoin stands out as one of the most effective therapies for moderate-to-severe acne vulgaris [8]. Oral isotretinoin is also used to manage mild to moderate acne refractory to topical and other oral therapy options [8]. The treatment dosage of isotretinoin depends on the severity of the acne. Treatment is generally initiated at a lower dose and can be adjusted based on the therapeutic response [8]. The two primary dosing strategies available include a standard regimen (0.5–1.0 mg/kg/day) and a low-dose regimen (<0.5 mg/kg/day) [8].

A population-based study revealed that 64% of first isotretinoin prescriptions were given to patients who had not previously used other anti-acne medications [15]. This study shows that isotretinoin usage is predominant among individuals between 13 and 45 years, with 50% of the participants being female [15]. The Food and Drug Administration (FDA) introduced the iPLEDGE Risk Evaluation and Mitigation Strategy (REMS) program for all patients receiving isotretinoin in 2006 to prevent teratogenic risks [16]. Despite the strict regulatory measures in the iPLEDGE program, an estimated 150 fetal exposure to isotretinoin are reported every year [17]. Additionally, isotretinoin can lead to dermatologically adverse effects. A systematic review indicated that approximately 65% of all reported side effects were related to dermatologic conditions [18]. Additional adverse effects that required monitoring included hyperlipidemia, elevated liver enzymes, depression, photophobia, and headache [18]. This highlights the need for a comprehensive assessment of patients’ knowledge about isotretinoin side effects and its proper use.

The majority of studies assessing isotretinoin in Saudi Arabia have primarily focused on evaluating the perceptions of the general public, investigating specific adverse effects, or assessing adherence to precautionary protocols. A study conducted on a sample of 3001 individuals from the general population in Saudi Arabia indicated that more than 50% had poor knowledge about the use of isotretinoin [19]. This is particularly concerning given the significant severity of the adverse effect profile associated with isotretinoin. Although one study included 356 dermatology clinic patients, participants in that research were recruited with the assistance of physicians and nurses and did not follow specific inclusion or exclusion criteria, which may have limited the accuracy and consistency of its responses [20]. In contrast, the present study targeted acne patients attending a dermatology clinic in the Qassim region, where a research team member was consistently present on-site to systematically recruit participants. This approach enhanced the reliability of patient-reported information and provides a novel perspective by capturing real-world experiences and awareness in a clinical setting. Additionally, we aimed to provide a more comprehensive survey of patients’ awareness of commonly reported adverse effects (e.g., teratogenicity, dryness, hepatotoxicity), adherence to precautionary measures, and experiences with the medication. By targeting acne patients directly, this study provides a focused evaluation of these critical aspects to help address gaps in patient education and counseling.

## 2. Materials and Methods

A cross-sectional study design was used to collect data from acne vulgaris patients through a self-administered questionnaire. This study was conducted in the dermatology outpatient clinics of a tertiary hospital in the Qassim region, Saudi Arabia, from December 2023 to February 2024. This study obtained ethical approval from the Regional Research Ethics Committee of Qassim Province, which is registered under the National Committee of Bioethics (NCBE) with Registration No. H-04-Q-001. The reporting of this cross-sectional study follows the Strengthening the Reporting of Observational Studies in Epidemiology (STROBE) guidelines to ensure methodological transparency and completeness.

The questionnaire used in this study was adopted after a review of previous studies published in the literature [20]. The questionnaire was then pretested on 10 patients with acne vulgaris to check the understanding and clarity of the questionnaire. The full questionnaire is included in the Supplementary Materials (Figure S1). The questionnaire consisted of 12 questions along with options for possible answers. This study specifically targeted patients in the Qassim region and implemented a rigorous methodology to enhance the reliability of the findings. A research team member was consistently present on-site at the dermatology clinic to systematically recruit patients with acne vulgaris and distribute the survey in person, thereby improving response accuracy and reducing selection bias.

The respondents were asked to select the most relevant response to each question. The questionnaire collected demographic data, such as age, gender, marital status, and educational background. The survey then assessed patients’ familiarity with isotretinoin and their sources of information, whether from healthcare professionals, personal experiences, social contacts, or online sources. The questionnaire also evaluated patients’ understanding of isotretinoin’s common side effects, such as skin dryness, changes in liver function, and potential risks during pregnancy. This study addressed patients’ practical experiences with isotretinoin, including the most significant side effects patients encountered. Finally, this study further analyzed the responses based on gender and education to identify variations in knowledge, concerns, and experiences with the medication. This approach provided valuable insights into how demographic factors influence patients’ understanding and use of isotretinoin.

### 2.1. Inclusion/Exclusion Criteria

This study included acne vulgaris patients who were 15 years of age or older, had a documented current or recent history of using isotretinoin therapy within the past 12 months, and provided informed consent to participate, in accordance with the ethical approval granted by the Regional Research Ethics Committee. The minimum age of 15 years was selected based on epidemiological evidence indicating that individuals aged 15–29 years represented the primary group prescribed isotretinoin for acne treatment [21]. Additionally, patients younger than 15 years may have a limited capacity to provide accurate and reliable self-reported information regarding their treatment experiences and perceptions. The 12-month cutoff for recent isotretinoin therapy was determined based on practical considerations to balance recall accuracy with the need to capture experiences among patients who had recently completed treatment.

### 2.2. Sample Size

In this study, a convenience sample size of 131 patients was employed due to practical considerations and the availability of participants.

### 2.3. Data Analysis

The returned data responses were entered and analyzed using Statistical Package for Social Sciences software (SPSS, version 26, SPSS, Chicago, IL, USA). Descriptive analysis was used for the categorical variables. The Chi-square test was used to find associations between the measured outcome and independent variables. A *p*-value of < 0.05 was used for any statistical significance associations obtained.

## 3. Results

### 3.1. Patients’ Demographic Characteristics

A total of 131 patients participated and completed the survey, with 108 (82.4%) being females and 23 (17.6%) being males. The majority of the respondents were in the 23–33 age group, and 102 (77.9%) of the participants had a university-level education, as illustrated in Table 1.

### 3.2. Patients’ Knowledge and Concerns About Isotretinoin

A total of 128 respondents (97.7%) reported having prior knowledge about isotretinoin. Among those who were knowledgeable about isotretinoin, 108 (82.4%) were female, and 23 (17.6%) were male. The difference in knowledge between the genders was found to be statistically significant (*p* = 0.024). The most commonly reported sources of information were colleagues, friends, or family members, followed by acne patients with prior isotretinoin use, and then healthcare professionals. When patients were asked to identify the common side effects of isotretinoin, dryness was recognized by 68 patients (51.9%), followed by liver function changes, reported by 32 patients (24.4%), and fetal abnormalities, mentioned by 17 patients (13%). Regarding patient concerns, the most prominent issue associated with starting isotretinoin was the potential for side effects, reported by 88 patients (67.2%). This was followed by concerns about the duration of treatment, noted by 12 patients (9.2%), and pregnancy-related risks, mentioned by 5 patients (3.8%). Further details are outlined in Table 2.

### 3.3. Patients’ Experience

This study documented a range of side effects reported by patients, with skin dryness being the most frequently mentioned. Furthermore, the data indicates that 33 patients (25.2%) had not attempted any other treatment prior to initiating isotretinoin therapy. Additionally, 20 patients (15.3%) reported that they were not tested for liver enzymes either before or during the course of treatment. Additional details are provided in Table 3.

### 3.4. Analysis of Knowledge, Concern Parameters, and Patients’ Experience with Isotretinoin

This study assessed knowledge, concerns, and patients’ experience with isotretinoin based on gender and education groups. Significant differences were found in several areas. Female patients with acne were generally more knowledgeable about isotretinoin, with 107 females (83.6%) having prior knowledge compared to 21 males (16.4%) (*p* = 0.024). Additionally, 102 females (84.3%) had a general understanding of potential side effects compared to 19 males (15.7%) (*p* = 0.05). Regarding concerns when initiating isotretinoin, female patients demonstrated significantly higher levels of concern, with 79 females (89.8%) particularly concerned about potential side effects compared to only 9 males (10.2%) (*p* < 0.001). Furthermore, a statistically significant difference was observed in the prior use of acne medications, with 108 females (82.4%) being more likely to have used other treatments compared to 23 males (17.6%) (*p* = 0.012). However, no significant associations were identified between gender and the source of information, specific side effects known by the patients, and the most frequently reported side effects, as detailed in Table 4 and Table 5.

Educational background also influenced the knowledge of side effects, as shown in Table 6. University-educated patients demonstrated a higher awareness (77.9%) compared to those with a school-level education (22.1%) (*p* = 0.003).

## 4. Discussion

This study assessed the knowledge, concerns, and experiences with isotretinoin among individuals with acne vulgaris. The findings were analyzed according to gender and education levels, providing critical insights into patient awareness and the use of this widely prescribed treatment for moderate to severe acne. A notable 97.7% of participants reported prior awareness of isotretinoin. However, the most frequently cited sources of information were non-healthcare professionals. The primary source of information identified in this study was colleagues, friends, or family members (37.4%), followed by previous users of isotretinoin (26.0%). Only 24.4% of participants received information from medical doctors or pharmacists. Similar results were observed in previous studies where nearly half of the patients relied on non-medical sources for information about isotretinoin [22,23]. These findings highlight a significant gap in counseling for patients with acne vulgaris, particularly those using isotretinoin. This gap is concerning given the well-documented risk profile of isotretinoin, including its pronounced teratogenic effects. This reliance on non-medical sources of information increases the likelihood of misinformation and the improper use of the medication, potentially leading to adverse outcomes. In this study, 92.4% of participants perceived themselves as generally knowledgeable about the potential side effects of isotretinoin. This result aligns with other studies that have shown acne patients often consider themselves well informed about isotretinoin [24,25]. However, our findings indicate that only 3.8% of participants recognized the teratogenic risks associated with its use, raising concerns about the sources of information patients rely on. The evidence suggests that poor adherence with contraceptive recommendations and prevention protocols among patients, pharmacists, and prescribers remains a global issue, which can lead to detrimental consequences such as unintended pregnancies and severe fetal abnormalities [26].

Healthcare providers should ensure equitable access to information and counseling for acne patients, particularly those prescribed isotretinoin. The high levels of burnout reported among dermatologists and pharmacists in various settings may contribute to reduced patient counseling and the inconsistent reinforcement of precautionary measures related to isotretinoin use [27,28]. In this study, female patients demonstrated significantly better knowledge of isotretinoin and its side effects than male patients with acne. Specifically, 83.6% of female participants were aware of isotretinoin, compared to only 16.4% of males (*p* = 0.024). Additionally, 84.3% of females had a general understanding of the medication’s potential side effects, such as dryness and liver function changes, whereas only 15.7% of males had similar knowledge (*p* = 0.05). This disparity may be attributed to female patients having more opportunities to access information about isotretinoin or being more proactive in seeking such information. The sample’s overrepresentation of university students and female participants may have influenced the results. University students are likely to demonstrate greater baseline knowledge, higher exposure to health education, and more proactive attitudes toward health-related topics compared to the general population. Similarly, female respondents tend to exhibit elevated health-seeking behaviors and utilization of health information, potentially leading to more aware or educated responses [29]. Consequently, these demographic biases may limit the generalizability of the findings to populations with a lower educational attainment or different gender distributions, highlighting the need for caution in broader interpretation.

This study also investigated side effect experiences among acne patients using isotretinoin. The most commonly reported side effects were skin dryness (45.8%) and dryness of the eyes and nose (34.4%). This is consistent with other studies that showed dryness being the most common adverse effect experienced with isotretinoin [23,25,30]. While these are well-documented side effects of isotretinoin, their high prevalence among the participants highlights the importance of educating patients on how to manage these adverse effects, such as using moisturizers and eye drops. Interestingly, 7.6% of patients reported no side effects, indicating variability in the tolerance to the medication.

Liver function monitoring is a key parameter of isotretinoin therapy due to its potential hepatotoxicity. In this study, 55.7% of patients reported undergoing liver function tests before starting isotretinoin and continuing them every three months during treatment, while 29% reported testing only before starting treatment. However, 15.3% of patients did not undergo any liver function testing, raising concerns about adherence to monitoring protocols. Another study by Imam et al. showed that an even higher percentage of participants, namely more than half of their participants, did not undergo liver function tests before isotretinoin therapy [30]. These findings highlight the need for healthcare providers to emphasize the importance of regular testing and follow-up to ensure the safe use of isotretinoin.

In conclusion, while this study demonstrates a high awareness of isotretinoin, significant gaps remain in knowledge, particularly among male patients, regarding the side effects and the importance of monitoring during treatment. Gender differences suggest that female patients are more engaged and concerned about the risks associated with isotretinoin use. However, further education and counseling are still needed, particularly regarding the teratogenic effects of isotretinoin.

### Strengths and Limitations of This Study

This study is one of the few to specifically assess the knowledge, concerns, and experiences of acne patients using isotretinoin, as well as the common side effects encountered. However, it has several limitations. First, this study relied on a cross-sectional design and self-reported data, which limits the ability to infer causality and may introduce social desirability bias. This potential bias may have led participants to respond to questions about their knowledge and practices in a way they believed was expected. Second, patients’ knowledge and behaviors regarding isotretinoin may change over time, which is an inherent limitation of the cross-sectional design. Third, the study population was predominantly female and highly educated (university students), introducing a potential selection bias that may limit the representativeness of the findings to the broader acne patient population. Fourth, this study had a relatively small sample size, which may limit the generalizability of the findings. Finally, although similar studies have been conducted in other countries, differences in cultural context, healthcare systems, and patient education practices may limit the direct comparability of the findings.

Despite these limitations, this study provides valuable insights and guidance. Our study specifically targeted acne vulgaris patients, which allowed for a more accurate representation of the sample. In contrast to prior investigations conducted in the general population or via online surveys, our approach ensured that only patients with clinically confirmed acne vulgaris who were receiving isotretinoin participated. The patients were recruited directly from dermatology clinics to strengthen the relevance and reliability of the findings for real-world clinical settings. Additionally, this offers important insights for health authorities in Saudi Arabia, highlighting the need to strictly enhance monitoring protocols for isotretinoin. Furthermore, this study emphasizes that healthcare providers should not assume that patients’ knowledge is always reliable, as their sources of information may not be accurate. Future studies could incorporate additional assessment methods, such as structured interviews or focus groups, to complement self-reported questionnaires and provide more in-depth insights. Additionally, future research should employ more rigorous sampling methods and larger sample sizes and consider multiple healthcare settings from different regions to improve the generalizability of the results.

## 5. Conclusions

This study highlights that the majority of acne patients demonstrate an adequate level of knowledge regarding isotretinoin and its adverse effects. The most frequently reported side effects were dryness of the skin, eyes, and nose. However, patients with acne require further counseling, particularly concerning the teratogenic risks and the importance of monitoring parameters during treatment. The primary sources of information about isotretinoin were non-medical, including colleagues, friends, or family members, followed by personal prior use and healthcare professionals such as physicians and pharmacists. A significant association was observed between patients’ educational levels and their knowledge of isotretinoin’s side effects, as well as their understanding of specific adverse reactions. This study also revealed significant associations between gender and both knowledge of isotretinoin and concerns about initiating treatment.

## Figures and Tables

**Table 1:** Demographic characteristics (*n* = 131).

Characteristic	*n* (%)
Gender	
Male	23 (17.6)
Female	108 (82.4)
Age groups (years)	
15–22	38 (29)
23–33	76 (58)
Marital status	
Non-married	98 (74.8)
Married	33 (25.2)
Level of education	
School	29 (22.1)
University	102 (77.9)

**Table 2:** Patients’ knowledge and concerns regarding isotretinoin (*n* = 131).

Questions	*n* (%)
Have you previously been informed about or heard of isotretinoin?	
Yes	128 (97.7)
No	3 (2.3)
What is your primary source of information regarding isotretinoin?	
Medical doctor or pharmacist	32 (24.4)
Acne patients who have used isotretinoin	34 (26.0)
Colleagues, friends, or family with no isotretinoin use	49 (37.4)
Internet or social media	12 (9.2)
Other	1 (0.7)
Not applicable	3 (2.3)
Are you generally knowledgeable about the potential side effects associated with isotretinoin?	
Yes	121 (92.4)
No	10 (7.6)
Which side effects related to isotretinoin do you recognize?	
Dryness	68 (51.9)
Changes in liver function test	32 (24.4)
Fetal Abnormalities	17 (13.0)
Constipation	4 (3.1)
Nothing	10 (7.6)
What is your primary concern regarding initiating treatment with isotretinoin?	
Side effects	88 (67.2)
The duration of usage	12 (9.2)
Pregnancy dangerous side effects	5 (3.8)
Nothing	26 (19.8)

**Table 3:** Patients’ experience with isotretinoin (*n* = 131).

Questions	*n* (%)
What was the most significant side effect you experienced during isotretinoin treatment?	
Skin dryness	60 (45.8)
Dryness of eyes and nose	45 (34.4)
Photosensitivity	8 (6.1)
Had no side effect	10 (7.6)
Others	8 (6.1)
Did you use any other acne medications before starting isotretinoin treatment?	
Topical acne cream	79 (60.3)
Topical antibiotics	19 (14.5)
Nothing	33 (25.2)
Was your liver function tested prior to or during isotretinoin treatment?	
Yes, only before starting	38 (29.0)
Yes, before starting and every 3 months	73 (55.7)
No testing	20 (15.3)

**Table 4:** Knowledge and concerns about isotretinoin among males and females (*n* = 131).

Questions	Male (%)	Female (%)	Total (%)	*p* Value *
Have you previously been informed about or heard of isotretinoin?	23 (17.6)	108 (82.4)	131 (100)	0.024 *
Yes	21 (16.4)	107 (83.6)	128 (97.7)	
No	2 (66.7)	1 (33.3)	3 (2.3)	
What is your primary source of information regarding isotretinoin?	23 (17.6)	108 (82.4)	131 (100)	NS
Medical doctor or pharmacist	6 (19.4)	25 (80.6)	32 (24.4)	
Acne patients who have used isotretinoin	2 (5.9)	32 (94.1)	34 (26.0)	
Colleagues, friends, or family with no isotretinoin use	9 (18.8)	39 (81.3)	49 (37.4)	
Internet or social media	4 (28.6)	10 (71.4)	12 (9.2)	
Other	0 (0.0)	1 (100.0)	1 (0.7)	
Not applicable	2 (66.7)	1 (33.3)	3 (2.3)	
Are you generally knowledgeable about the potential side effects associated with isotretinoin?	23 (17.6)	108 (82.4)	131 (100)	0.05 *
Yes	19 (15.7)	102 (84.3)	121 (92.4)	
No	4 (40.0)	6 (60.0)	10 (7.6)	
Which side effects related to isotretinoin do you recognize?	23 (17.6)	108 (82.4)	131 (100)	NS
Dryness	14 (20.6)	54 (79.4)	68 (51.9)	
Fetal abnormalities	1 (5.9)	16 (94.1)	17 (13.0)	
Changes in liver function test	3 (9.4)	29 (90.6)	32 (24.4)	
Constipation	1 (25.0)	3 (75.0)	4 (3.1)	
Nothing	4 (40.0)	6 (60.0)	10 (7.6)	
What is your primary concern regarding initiating treatment with isotretinoin?	23 (17.6)	108 (82.4)	131 (100)	<0.001 *
Side effects	9 (10.2)	79 (89.8)	88 (67.2)	
The duration of usage	8 (66.7)	4 (33.3)	12 (9.2)	
Pregnancy dangerous side effects	0 (0.0)	5 (100.0)	5 (3.8)	
Nothing	6 (23.1)	20 (76.9)	26 (19.8)	

* Significant; NS = not significant.

**Table 5:** Patients’ experience with isotretinoin among males and females (*n* = 131).

Questions	Male (%)	Female (%)	Total (%)	*p* Value *
What was the most significant side effect you experienced during isotretinoin treatment?	23 (17.6)	108 (82.4)	131 (100)	NS
Skin dryness	14 (23.3)	46 (76.7)	60 (45.8)	
Dryness of eyes and nose	8 (17.8)	37 (82.2)	45 (34.4)	
Photosensitivity	0 (0.0)	8 (100.0)	8 (6.1)	
Had no side effect	0 (0.0)	10 (100.0)	10 (7.6)	
Others	1 (12.5)	7 (87.5)	8 (6.1)	
Did you use any other acne medications before starting isotretinoin treatment?	23 (17.6)	108 (82.4)	131 (100)	0.012 *
Topical acne cream	8 (10.1)	71 (89.9)	79 (60.3)	
Topical antibiotics	4 (21.1)	15 (78.9)	19 (14.5)	
Nothing	11 (33.3)	22 (66.7)	33 (25.2)	
Was your liver function tested prior to or during isotretinoin treatment?	23 (17.6)	108 (82.4)	131 (100)	NS
Yes, only before starting	4 (10.5)	34 (89.5)	38 (29.0)	
Yes, before starting and every 3 months	13 (17.8)	60 (82.2)	73 (55.7)	
No testing	6 (30.0)	14 (70.0)	20 (15.3)	

* Significant; NS = not significant.

**Table 6:** Comparison of knowledge about isotretinoin by education level among participants (*n* = 131).

Questions	School (%)	University (%)	Total (%)	*p* Value *
Are you generally knowledgeable about the potential side effects associated with isotretinoin?	29 (22.1)	102 (77.9)	131 (100)	0.003 *
Yes	23 (19.0)	98 (81.0)	121 (92.4)	
No	6 (60.0)	4 (40.0)	10 (7.6)	
Which side effects related to isotretinoin do you recognize?	29 (22.1)	102 (77.9)	131 (100)	<0.001 *
Dryness	12 (17.6)	56 (82.4)	68 (51.9)	
Fetal abnormalities	0 (0.0)	17 (100.0)	17 (13.0)	
Changes in liver function test	8 (25.0)	24 (75.0)	32 (24.4)	
Constipation	3 (75.0)	1 (25.0)	4 (3.1)	
Nothing	6 (60.0)	4 (40.0)	10 (7.6)	

* Significant.

## Data Availability

The original contributions presented in this study are included in the article/Supplementary Material. Further inquiries can be directed to the corresponding author.

## Supplementary Materials

The following supporting information can be downloaded at https://www.mdpi.com/article/10.3390/healthcare13131617/s1, Figure S1: Questionnaire to Measure Patients’ Awareness of the Use of Roaccutane (isotretinoin).
