# Supplementary material for: Adverse Effects and Precautionary Measures for Isotretinoin Use in Patients with Acne Vulgaris: A Single-Center Study
Source: Healthcare (Basel). 2025 Jul 7;13(13):1617. doi: 10.3390/healthcare13131617 (PMC12250291; doi:10.3390/healthcare13131617)
Supplement: Supplementary file 1 [file healthcare-13-01617-s001.zip › healthcare-3712504-supplementary.pdf]

استبيان لقياس وعي المرضى حول استخدام حبوب الروكتان (isotretinoin)

Figure S1. Questionnaire to Measure Patients' Awareness of the Use of Roaccutane (isotretinoin)

**Consent to Participate in the Study:**	**أولاً: الموافقة على المشاركة في الدراسة:**
In this questionnaire, we aim to measure patients' awareness of the medication Roaccutane (isotretinoin), which is used to treat acne vulgaris	في هذا الاستبيان نهدف لقياس مدى وعي المرضى عن دواء الروكتان (isotretinoin) والذي يستخدم لعلاج حب الشباب
We invite you to participate in this research study aimed at measuring patients' awareness of Roaccutane (isotretinoin) and its use in treating acne vulgaris. Your participation is entirely voluntary, and you have the right to withdraw at any time without any consequences. The information collected will be kept confidential and used solely for research purposes.<br><br>By completing this questionnaire, you agree to participate in this study and allow your responses to be used for research analysis. No identifying personal information will be recorded, and your anonymity will be maintained throughout the study.<br><br>If you have any questions regarding the study, feel free to contact the research team (0558-373-999).<br><br>Thank you for your valuable contribution.<br><br>Please indicate your choice below:<br>☐ I agree to participate in the study<br>☐ I do not agree to participate in the study	ندعوك للمشاركة في هذه الدراسة البحثية التي تهدف إلى قياس وعي المرضى حول دواء الروكتان (ايزوتريتينوين) واستخدامه في علاج حب الشباب. مشاركتك طوعية بالكامل، ويحق لك الانسحاب في أي وقت دون أي عواقب. سيتم الحفاظ على سرية المعلومات التي يتم جمعها ولن تُستخدم إلا لأغراض البحث فقط.<br><br>بإكمال هذا الاستبيان، فإنك توافق على المشاركة في هذه الدراسة وتسمح باستخدام إجاباتك للتحليل البحثي. لن يتم تسجيل أي معلومات شخصية تُعرّف بك، وسيتم الحفاظ على خصوصيتك طوال فترة الدراسة.<br><br>إذا كانت لديك أي أسئلة حول الدراسة، لا تتردد في التواصل مع فريق البحث (0558-373-999).<br><br>شكرًا لمساهمتك القيمة.<br><br>يرجى تحديد اختيارك أدناه<br>☐ أوافق على المشاركة في الدراسة<br>☐ لا أوافق على المشاركة في الدراسة
Signature:	التوقيع:
Date:	التاريخ:

ثانياً: المعلومات الشخصية

1. **Gender:**<br>☐ Male<br>☐ Female	1. **الجنس:**<br>☐ ذكر<br>☐ أنثى
2. **Age:**<br>☐ 15-22 years<br>☐ 23-33 years<br>☐ 34-60 years<br>☐ Above 60 years	2. **العمر:**<br>☐ 22-15 سنة<br>☐ 33-23 سنة<br>☐ 60-34 سنة<br>☐ فوق 60 سنة
**3. Marital status:**<br>☐ Single<br>☐ Married	3. **الحالة الإجتماعية:**<br>☐ أعزب/عزباء<br>☐ متزوج/متزوجة
**4. Education Level:**<br>☐ Elementary<br>☐ Intermediate<br>☐ High school<br>☐ University	4. **مستوى التعليم:**<br>☐ ابتدائي<br>☐ متوسط<br>☐ ثانوي<br>☐ جامعي

ثالثاً: الإستبيان

**5. Have you heard about Roaccutane (isotretinoin) pills before?**<br>☐ Yes<br>☐ No	5. **هل سمعت عن حبوب الروكتان (isotretinoin) من قبل؟**<br>☐ نعم<br>☐ لا
**6. What are your sources of knowledge about Roaccutane (isotretinoin)?**<br>☐ Doctor or Pharmacist<br>☐ Acne patient who have used Roaccutane<br>☐ A colleague, friend, or family member<br>☐ From the internet or social media<br>☐ Other<br>☐ Not applicable	6. **ماهي مصادر معرفتك للروكتان (isotretinoin)؟**<br>☐ الطبيب أو الصيدلي<br>☐ من تجربة سابقة لمريض استخدم الروكتان<br>☐ من زميل أو صديق أو أحد أفراد العائلة<br>☐ من الإنترنت ووسائل التواصل الاجتماعي<br>☐ أخرى<br>☐ لا ينطبق
**7. Do you know the side effects of Roaccutane?**<br>☐ Yes<br>☐ No ** If you choose this option, please answer the next question (No. 8) by selecting the last option (Not applicable)	7. **هل تعرف/تعرفين الأضرار الجانبية للروكتان؟**<br>☐ نعم<br>☐ لا **في حال إجابة هذا الخيار يرجى إجابة السؤال التالي (رقم 8) بالاختيار الأخير (لا ينطبق)
**8. What side effects do you know about Roaccutane (isotretinoin)?**<br>☐ Dryness<br>☐ Fetal abnormalities<br>☐ Effect on liver functions<br>☐ Constipation<br>☐ Not applicable	8. **ماهى الأعراض الجانبية التى تعرفها عن الروكتان؟**<br>☐ الجفاف<br>☐ تشوهات الأجنة<br>☐ تأثيره على وظائف الكبد<br>☐ الإمساك<br>☐ لا ينطبق
**9. Are you willing to use Roaccutane?**<br>☐ Yes<br>☐ No<br>☐ I do not have acne (If your answer is this option, please select (Not applicable) for the next four questions (10, 11, 12, 13)	9. **هل أنت مستعد لاستخدام الروكتان (isotretinoin)؟**<br>☐ نعم<br>☐ لا<br>☐ لست مصابا" بحب الشباب (في حال كانت اجابتك بهذا الخيار، يرجى اختيار (لا ينطبق) لاجابة الأسئلة الاربعة التالية (10,11,12,13).

**10. What was the primary concern before start using Roaccutane? (select all that apply)?**<br>☐ Fear of side effects<br>☐ Duration of use<br>☐ fetal abnormalities<br>☐ Nothing<br>☐ Not applicable	**10. ماهو أكبر سبب أقلقك عند البدء باستخدام الروكتان (خيار متعدد)**<br>الخوف من الأعراض الجانبية ☐<br>مدة الإستخدام ☐<br>الآثار الجانبية الخطيرة على الحامل (تشوهات الأجنة) ☐<br>لا شيء ☐<br>لا ينطبق ☐
**11. What was the most significant side effect you experienced? (select all that apply)**<br>☐ Dry skin<br>☐ Dry lips and nose<br>☐ Sunlight sensitivity<br>☐ No side effects experienced<br>☐ Other<br>☐ Not applicable	**11. ماهو أكثر عرض جانبي عانيت منه؟ (خيار متعدد)**<br>جفاف الجلد ☐<br>جفاف الشفتين والفم ☐<br>الحساسية من ضوء الشمس ☐<br>لم يكن هناك أي آثار جانبية ☐<br>أخرى ☐<br>لا ينطبق ☐
**12. Did you perform liver function tests before starting the treatment and every 3 months after?**<br>☐ Yes, only before starting the treatment<br>☐ Yes, before starting the treatment and every 3 months<br>☐ No, I did not test for liver enzymes<br>☐ Not applicable	**12. هل قمت بتحليل وظائف الكبد قبل البدء بالعلاج وكل 3 شهور بعد بدايته؟**<br>نعم قبل البدء بالعلاج فقط ☐<br>نعم قبل البدء بالعلاج وتابعت كل 3 أشهر ☐<br>لا لم أقم بتحليل وظائف الكبد ☐<br>لا ينطبق ☐
**13. Have you tried other treatments for acne before starting Roaccutane? If yes, what are they?**<br>☐ Topical exfoliating creams<br>☐ Topical antibiotic solutions<br>☐ No, I have not tried<br>☐ Other<br>☐ Not applicable	**13. هل حاولت تجربة علاجات أخرى لحب الشباب قبل البدء بالروكتان وماهي؟**<br>كريمات التقشير الموضعية ☐<br>محاليل المضادات الحيوية الموضعية ☐<br>لا لم أجرب من قبل ☐<br>أخرى ☐<br>لا ينطبق ☐
